# Tobacco smoking and blood parameters in the kurdish population of Iran

**DOI:** 10.1186/s12872-023-03433-2

**Published:** 2023-08-14

**Authors:** Ebrahim Shakiba, Mehdi Moradinazar, Zohreh Rahimi, Farid Najafi, Yahya Pasdar, Maryam Kohsari

**Affiliations:** Behavioral disease Research Center, Kermanshah University of Medical Sciences, Kermanshah, Iran

**Keywords:** Smoking, Red blood cell indices, White blood cell indices, Platelet indices

## Abstract

**Objective:**

This study investigates the relationship between smoking and blood parameters in the Iranian Kurdish population.

**Method:**

The current study was conducted based on the recruitment phase of the Ravansar Non-Communicable Disease (RaNCD) cohort study.

**Results:**

Current smokers had higher levels of RBC count, HCT, HGB, MCV, MCH, MCHC, WBC count, and GR%, than in other groups significantly. Passive smokers had higher levels of PLT count and PCT statistically. The increasing exposure time of smoking positively affected WBC count, GR%, PLT count, PCT, and RDW in female passive smokers. In addition, heavy smokers, as well as participants with a higher duration time of smoking, had the same results for significantly lower levels of lymphocyte and monocyte and a higher level of RBC indices.

**Conclusion:**

According to the present study, along with the current smokers, the intensity of smoking, as well as the duration time of the smoke, could have a positive correlation with blood parameters. Furthermore, passive smokers and specifically secondhand female smokers were more vulnerable to smoke.

## Introduction

Various studies have illustrated that unhealthy lifestyles, inactivity, poor nutrition, and smoking are the main risk factors for public health [1]. Smoking is the most preventable account of death globally [2]. Cigarette smoke ingredients, including carbon monoxide, hydrogen cyanide, benzene, nitrosamines, and nicotine, are toxic and, cause damage to the body in several ways [3]. Smokers have a higher chance of developing cardiovascular disease, cancers, and pulmonary diseases [1]. According to WHO, tobacco-related death will reach 8 million dying in 2030 [4]. Besides, the harmful effect of smoking is not limited to active tobacco smokers. Evidence represents that passive smokers are susceptible to the detrimental effects of cigarette smoke as much as active smokers [5] and 0.9 million smoking-related deaths annually belong to passive smokers [5, 6]. A meta-analysis study reported that 20% of Iranian males are smoking [7]. Another study in 2016 on the Iranian adult population demonstrated that the prevalence of current tobacco use for men and women was 24.4% and 3.8%, respectively [8]. Some evidence illustrated the increasing smoking rate among young Iranian adults [9].

In studies on the health effects of smoking, various investigations have examined the impact of tobacco on end products of hematopoiesis [3, 6, 10, 11]. However, the impact of smoking on hematopoiesis end products has not been adequately identified, and the results are contradictory. It is still questionable whether cigarette smoking impacts the hematopoietic system or not [10]. Some studies have shown that smoking increases red blood cell (RBC) count, hematocrit (HCT), hemoglobin (HGB), mean corpuscular volume (MCV) and mean corpuscular hemoglobin (MCH) [10]. Some investigations did not find any difference in RBC indices between smokers and non-smokers [6]. There are also conflicting results for white blood cells(WBC) and platelets(PLT) [10].

On the other hand, information in this regard is very limited on the Iranian population. Therefore, in the present study, we examined the blood parameters in five smoking status categories (secondhand smoke, current smokers, regular age of onset smoke, smoking intensity, and smoking duration) from the baseline data of the RaNCD study on the Kurdish population in the west of Iran.

## Method

### Population of study

The current study emanated from the baseline data of the Ravansar Non-Communicable Disease (RaNCD) cohort study conducted on the Kurdish population in the west of Iran. The RaNCD cohort study is a part of the Prospective Epidemiological Research Studies (PERSIAN) in Iran, and the initial phase was from 2014 to 2017. Further details of the protocol and research guide exist at www.persiancohort.com. The Ethics Committee of the Deputy of Research and Technology of Kermanshah University of Medical Sciences has approved this study. Participants entered the investigation after being aware of the study process and signed a written consent [12].

### Smoking habit

National Health Insurance Scheme (NHIS) was used to assess smoking habits. The subjects were classified into four groups: smokers, non-smokers, former smokers, and passive smokers. Smokers are defined as individuals who had smoked at least 100 cigarettes. The non-smoker group included those who had not smoked at least 100 cigarettes during their lifetime. Individuals that have a history of smoking at least 100 cigarettes were defined as former smokers. Passive smokers referred to the participants who inhale the smoke of cigarettes from other people [13, 14]. Heavy smokers are defined as those who smoke ≥ 25 cigarettes per day [15] and light smokers were considered as individuals that smoke < 10 cigarettes per day [16].

The BIA device (InBody 770 BIOSPACE, Korea) measured the weight. A 0.1 precision stadiometer measured the height of the participants. The Body Mass Index (BMI) was derived by dividing weight (kg) by squaring the height (m2). Based on the 24-h physical activity and a 22-item questionnaire that considered work and leisure time in one week by participants, physical activity was defined on the METs/hour/day. The quality of nutrition was assessed according to the Healthy Eating Index − 2015 (HEI-2015), which evaluates 13 food groups. The HEI score is between 0 and 100, and a high score exhibits a better quality of nourishment [12]. The Joint National Committee on Prevention, Detection, Evaluation, and Treatment of High blood pressure (JNC-7) was applied to diagnose hypertension. This study considered participants with a systolic blood pressure(SBP) ≥ 140 mm Hg and/or diastolic blood pressure (DBP) ≥ 90 mmHg and/or those with current use of antihypertensive drugs as hypertensive [12]. Cardiovascular disease was defined based on self-report and a history of taking cardiovascular drugs.

### Excluded criteria

The participants with disorders that might affect the hematological parameters like pregnancy, active gastrointestinal bleeding, liver disorders such as non-alcoholic fatty liver disease (NAFLD), HBV, and HCV, also participants with coagulation disorders and anemia, cancer, type 2 diabetes mellitus, and also kidney and thyroid disorders were excluded from the investigation. From 10,056 participants in the RaNCD cohort study, 8736 subjects were eligible to enter the study.

### Blood analysis

All blood samples were taken by venoject and then transferred to CBC tubes containing EDTA. RBC indices, including (RBC count, HCT, HGB, MCV, MCH, mean corpuscular hemoglobin concentration (MCHC), red cell distribution width (RDW-CV)), WBC indices, including (WBC count, lymphocyte, monocyte, and granulocyte percent (GR %)), and platelet indices, including (PLT count, plateletcrit (PCT), and platelet distribution width (PDW)) were measured by a Sysmex cell counter.

### Statistical analysis

T-test, one-way analysis of variance (ANOVA), and chi-square test, respectively used to analyze the quantitative and qualitative variables. The quantitative data were presented with mean and standard division (SD) and qualitative data were shown with percentages and frequencies. The association between blood parameters and different smoking categories was evaluated via a multivariable linear regression model. For a more acceptable assessment, the model adjusted for possible confounding variables such as age, gender, alcohol consumption, cardiovascular disorder (CVD), BMI, MET, HEI, and blood pressure (SBP and DBP). The relationship between smoking exposure time and blood parameters based on gender was represented by linear fit plot prediction. A p-value < 0.05 had considered statistically significant. The data analyses were done by Stata software (version 14.2) (Stata Corp, College Station, TX, USA).

## Results

Table 1. Presented the general characteristic of the participants. The mean age of the total participant was 47.2 ± 8.2 years and 4343 (49.7%) were men. The prevalence of CVD in the study population was 15.5%.

**Table 1:** General characteristics of the participants

Variables	male %	Age (years)	MET (hour/day)	Alcohol%	CVD%	BMI (kg/m^2^)	SBP (mmHg)	DBP (mmHg)	Healthy eating index			
									**Q1**	**Q2**	**Q3**	**Q4**
Total (8736)	4343(49.7)	47.2 ± 8.2	1.9 ± 0.7	449(5.1)	1361(15.5)	27.1 ± 4.6	108.1 ± 17	69.8 ± 9.9	2607(30)	2217(25.5)	2072(23.8)	180(20.7)
smoking status												
no	**1358(38.4)** ^*^	**46.4 ± 8.3** ^*^	**1.8 ± 0.6** ^*^	**65(14.7)** ^*^	**569(42.1)** ^*^	**27.4 ± 4.6** ^*^	**107.8 ± 17.1** ^*^	**69.3 ± 9.8** ^*^	925(26.2)	872(24.7)	886(25.1)	**845(24)** ^*^
current	968(92.1)	47.9 ± 7.9	2 ± 0.8	197(44.6)	116(8.6)	25.3 ± 4.5	106 ± 16.3	68.9 ± 9.4	450(42.9)	250(23.8)	235(22.4)	115(10.9)
former	639(83.3)	51.3 ± 7.9	1.9 ± 0.7	98(22.3)	174(13)	26.9 ± 4.8	112.7 ± 17.8	72.3 ± 10.7	246(32.1)	206(26.9)	165(21.5)	149(19.5)
passive	1377(41.9)	46.8 ± 8	1.9 ± 0.7	82(18.5)	502 (36.4)	26.9 ± 4.8	108 ± 16.8	70 ± 9.8	970 (29.6)	868(26.5)	766(23.4)	673(20.5)
Second hand smoke in current smokers												
No	**1358(38.4)** ^*^	**46.4(8.3)** ^*^	1.8 ± 0.6	**65(1.8)** ^*^	568(16.1)	27.4 ± 4.6	**107.8 ± 17.1** ^*^	**69.6 ± 9.8** ^*^	925(26.2)	871(24.7)	884(25.1)	845(24)
< 2 h	1120(42.4)	46.8(8)	1.9 ± 0.7	63(2.4)	401(15.2)	27.3 ± 4.6	107.6 ± 16.7	69.6 ± 9.8	780(29.6)	703(26.7)	615(23.4)	535(20.3)
2–5 h	206(38.3)	47.1(7.9)	1.9 ± 0.7	12(2.2)	84(15.6)	27.9 ± 4.6	110.7 ± 17.8	72 ± 10.1	159(29.8)	137(25.6)	128(24)	110(20.6)
> 5 h	49(45.3)	44.8(7.5)	1.9 ± 0.7	7(6.5)	8(7.5)	27.6 ± 4.2	105.3 ± 13.8	68.9 ± 8.8	30(27.8)	28(25.9)	25(23.1)	25(23.2)
Regular age of onset smoke												
No	**1358(38.4)** ^*^	**46.4 ± 8.3** ^*^	**1.8 ± 0.6** ^*^	**65(1.8)** ^*^	**569(16.1)** ^*^	**27.4 ± 4.6** ^*^	**10.7.8 ± 17.1** ^*^	69.3 ± 9.8	925(26.2)	872(24.7)	886(25.1)	**845(24)** ^*^
< 20 years	412(94.2)	48.4 ± 7.8	2 ± 0.7	79(18.1)	50(11.4)	24.9 ± 4.3	106 ± 16.5	68.8 ± 9.4	217(49.6)	99(22.7)	91(20.8)	30(6.9)
21–29 years	395(94.5)	47 ± 7.8	1.9 ± 0.8	93(22.2)	38(9.1)	25.2 ± 4.6	105.7 ± 15.3	68.6 ± 8.9	161(38.5)	103(24.6)	94(22.4)	60(14.3)
> 30 years	158(81.9)	48.6 ± 8.4	1.9 ± 0.7	25(13)	28(15)	26.1 ± 4.4	106.7 ± 17.7	69.5 ± 10.4	71(37)	48(25)	48(25)	25(13)
Smoking intensity in current smokers												
No	**1,358(38.4)** ^*^	**46.4 ± 8.3** ^*^	**1.8 ± 0.6** ^*^	**65(14.7)** ^*^	**569(42.1)** ^*^	**27.4 ± 4.6** ^*^	**107.8 ± 17.1** ^*^	69.3 ± 9.8	925(26.2)	872(24.7)	886(25.1)	**845(23.9)** ^*^
Light	256(84.5)	46.7 ± 8.4	1.8 ± 0.7	197(44.6)	116(8.6)	26.1 ± 4.1	106.7 ± 15.6	69.2 ± 9.1	103(34)	68(22.4)	83(27.4)	49(16.2)
Moderate	175(93)	47.2 ± 7.9	2 ± 0.8	98(22.8)	174(12.9)	25.3 ± 4.7	106.1 ± 16.1	68.8 ± 9.3	76 (40.4)	47(25)	42(22.3)	23(23.2)
Heavy	537(95.9)	48.8 ± 7.6	2 ± 0.8	82(18.5)	492(36.4)	24.8 ± 4.5	105.7 ± 16.7	68.7 ± 9.6	271(48.5)	135(24.1)	110(20)	43(7.7)
Smoking duration												
N0	**1,358(38.4)** ^*^	**46.4 ± 8.3** ^*^	**1.8 ± 0.6** ^*^	**65(24.8)** ^*^	**569(83.1)** ^*^	**24.4(4.6)** ^*^	**107.8 ± 17.1** ^*^	69.3 ± 9.8	925(26.2)	872(24.7)	886(25.1)	**845(24)** ^*^
0–4 years	47(79.7)	45.2 ± 8	1.9(0.8)	8(3)	7(1)	26.9(4.6)	105.6 ± 16.9	70.6 ± 10.3	24(41.4)	16(27.6)	13(22.4)	5(8.6)
5–14 years	119(93)	42.9 ± 7.4	2(0.7)	30(11.4)	8(1.2)	26.3(4.1)	105.2 ± 13.6	68 ± 8.7	36(28.1)	31(24.2)	40(32)	21(16.4)
> 15 years	802(92.8)	48.8 ± 7.7	2(0.8)	159(60.7)	101(14.7)	25(4.5)	106.2 ± 16.6	68.9 ± 9.4	390(45.1)	203(23.5)	182(21)	89(10.3)

*P-value < 0.05 (all the significant p-value are bolded in table)

As shown in Table 1, based on smoking status, the highest rate of current (92.1%) and former (83.3%) smokers have significantly belonged to the male population. Versus, the female population had a significantly higher percentage of passive smokers (58.1%). Also, the current smoker subjects dramatically had the highest rate of alcohol use (44.6%) and the lowest BMI (25.3 ± 4.5). Nevertheless, by 36.4%, passive smokers had a higher CVD prevalence than current and former smokers. Also, the former smoker group had significantly higher SBP and DBP. In the secondhand smoke category, the increased cigarette exposure time leads to increasing BMI. The highest prevalence of CVD was in participants exposed to secondhand smoke for less than two hours or between 2 and 5 h per day. Also, individuals with exposure between 2 and 5 h per day had the highest blood pressure. The female population in the age group over 30 has the highest rate of age onset of smoking (18.1%). In addition, participants with onset smoking over 30 years old; have the highest prevalence of CVD and increased BMI. There was a similar result about heavy smokers, which had the highest prevalence of CVD among smoker subjects. However, heavy smokers had the lowest SBP. In the smoking duration category, individuals using cigarettes over 15 years had a higher SBP than other smokers. Besides, this group had the highest rate of alcohol use (60.7%) and CVD prevalence (14.7%) among smokers.

As shown in Table 2. The current smokers have a significantly higher RBC count, HCT, HGB, MCV, MCH, MCHC, WBC count, Monocyte, PLT count, and PDW than other groups. The levels of HGB, MCHC, PLT count, and PCT dramatically increased with the increasing duration of cigarette smoke exposure in secondhand smokers.

**Table 2:** The mean level of the blood parameters is based on smoking categories. *P-value < 0.05 (all the significant p-value are bolded in table)

Variables	RBC count	HCT	HGB	MCV	MCH	MCHC	RDW	WBC count	LYM	Mono	GR%	PLT count	PCT	PDW
Total	4.9 ± 0.5	39.6 ± 4.1	14.2 ± 1.5	80.6 ± 7.0	28.9 ± 3.0	35.8 ± 1.4	11.0 ± 0.9	6.3 ± 1.5	41.2 ± 8.7	3.5 ± 1.2	55.2 ± 9.3	252.9 ± 61.6	0.1 ± 0.2	16.8 ± 0.8
smoking status														
no	**4.8 ± 0.5** ^*^	**39 ± 4** ^*^	**13.9 ± 1.5** ^*^	**80.3 ± 7** ^*^	**28.7 ± 2.9** ^*^	**35.7 ± 1.4** ^*^	11 ± 0.9	**6.3 ± 1.5** ^*^	41.3 ± 8.7	**3.4 ± 1.3** ^*^	55.1 ± 9.3	**257.9 ± 61.2** ^*^	0.1 ± 0.04	**16.8 ± 0.8** ^*^
current	5.1 ± 0.5	41.8 ± 3.7	15.1 ± 1.4	82.2 ± 7.4	29.9 ± 3.2	36.3 ± 1.3	10.9 ± 0.9	7.1 ± 1.9	40.9 ± 9.2	3.5 ± 1.2	55.5 ± 9.8	236.3 ± 53.8	0.1 ± 0.03	16.8 ± 0.8
former	5.1 ± 0.5	41.2 ± 3.9	14.8 ± 1.5	81.2 ± 6.8	29.2 ± 2.9	35.9 ± 1.4	11 ± 0.8	6.1 ± 1.4	41 ± 8.4	3.6 ± 1.2	55.2 ± 9	236.8 ± 56.9	0.2 ± 0.7	16.9 ± 0.8
passive	4.9 ± 0.5	39.2 ± 4.1	14 ± 1.5	80.3 ± 6.8	28.8 ± 3	35.8 ± 1.4	11 ± 0.9	6.3 ± 1.4	41.2 ± 8.6	3.5 ± 1.2	55.1 ± 9.2	**256.5 ± 63.6**	0.1 ± 0.05	16.8 ± 0.07
Secondhand smoke in current smokers														
No	**4.8 ± 0.5** ^*^	39 ± 4	**13.9 ± 1.5** ^*^	80.3 ± 6.9	28.7 ± 2.9	**35.7 ± 1.4** ^*^	11 ± 0.9	6.3 ± 1.5	41.3 ± 8.7	3.4 ± 1.3	55.1 ± 9.3	**257.9 ± 61.2** ^*^	**0.1 ± 0.04** ^*^	16.8 ± 0.8
< 2 h	4.8 ± 0.5	39.2 ± 4.1	14 ± 1.5	80.4 ± 6.9	28.9 ± 3	35.8 ± 1.5	10.9 ± 0.9	6.2 ± 1.4	41.1 ± 8.5	3.5 ± 1.2	55.2 ± 9.1	254.6 ± 62.8	0 0.1 ± 0.05	16.8 ± 0.8
2–5 h	4.9 ± 0.5	39 ± 3.9	14 ± 1.5	80 ± 6.5	28.8 ± 2.8	36 ± 1.3	11 ± 0.9	6.3 ± 1.5	41.4 ± 8.8	3.5 ± 1.2	55 ± 9.5	262.5 ± 64.6	0 0.2 ± 0.05	16.9 ± 0.7
> 5 h	4.9 ± 0.6	39.2 ± 4.4	14.1 ± 1.7	79.6 ± 6.8	28.7 ± 3	36 ± 1.3	11 ± 0.9	6.4 ± 2.1	42.4 ± 9	3.5 ± 1	54 ± 9.6	269.9 ± 73.7	0.2 ± 0.04	16.7 ± 0.8
Regular age of onset smoke														
No	**4.8 ± 0.5** ^*^	**39 ± 4** ^*^	**13.9 ± 1.5** ^*^	**80.3 ± 7** ^*^	**28.7 ± 2.9** ^*^	**35.7 ± 1.4** ^*^	**11 ± 0.9** ^*^	6.3 ± 1.5	**41.3 ± 8.7** ^*^	3.4 ± 1.3	55.1 ± 9.3	**257.9 ± 61.2** ^*^	**0.1 ± 0.04** ^*^	16.8 ± 0.8
< 20 years	5.1 ± 0.5	42 ± 3.7	15.2 ± 1.4	82.8 ± 7.9	30.1 ± 3.4	36.3 ± 1.4	10.9 ± 0.8	7.2 ± 1.9	40.1 ± 9.3	3.4 ± 1.1	56.3 ± 9.9	232.6 ± 51.5	0.1 ± 0.03	16.9 ± 0.8
21–29 years	5.1 ± 0.6	41.9 ± 3.5	15.2 ± 1.3	82.1 ± 7.4	29.8 ± 3.2	36.3 ± 1.3	11 ± 0.9	7.1 ± 1.9	41.4 ± 9.2	3.5 ± 1.2	55 ± 9.8	237.4 ± 54.3	0 0.1 ± 0.04	16.8 ± 0.7
> 30 years	5 ± 0.5	41 ± 4	14.9 ± 1.5	81.2 ± 6.1	29.5 ± 2.8	36.2 ± 1.2	10.9 ± 0.8	6.8 ± 1.8	41.5 ± 8.9	3.5 ± 1.2	54.9 ± 9.6	242.3 ± 57.3	0.1 ± 0.04	16.8 ± 0.7
Smoking intensity in current smokers														
No	**4.8 ± 0.5** ^*^	**39 ± 4** ^*^	**13.9 ± 1.5** ^*^	**80.3 ± 7** ^*^	**28.7 ± 2.9** ^*^	**35.7 ± 1.4** ^*^	11 ± 0.9	**6.3 ± 1.5** ^*^	**41.3 ± 8.7** ^*^	**3.4 ± 1.3** ^*^	55.1 ± 9.3	**257.9 ± 61.2** ^*^	**0.1 ± 0.04** ^*^	16.8 ± 0.8
Light	5 ± 0.5	41.1 ± 3.6	14.8 ± 1.4	81.3 ± 7.1	29.3 ± 3.1	36 ± 1.3	10.9 ± 0.9	6.4 ± 1.6	42.1 ± 8.8	3.5 ± 1.3	54.2 ± 9.5	232.5 ± 54.2	0.1 ± 0.04	16.9 ± 0.8
Moderate	5.1 ± 0.6	41.7 ± 3.4	15 ± 1.3	81.8 ± 7.9	29.6 ± 3.4	36.1 ± 1.4	10.9 ± 0.8	7.1 ± 2	42.1 ± 9.5	3.6 ± 1.1	54.2 ± 10.1	242.1 ± 53.5	0.1 ± 0.04	17 ± 0.7
Heavy	5.1 ± 0.5	42.2 ± 3.8	15.3 ± 1.4	82.8 ± 7.3	30.2 ± 3.1	36.4 ± 1.3	10.9 ± 0.9	7.5 ± 1.9	39.9 ± 9.2	3.4 ± 1.1	56.6 ± 9.8	236.5 ± 53.7	0.1 ± 0.03	16.8 ± 0.8
Smoking duration														
N0	**4.8 ± 0.5** ^*^	**39 ± 4** ^*^	**13.9 ± 1.5** ^*^	**80.3 ± 7** ^*^	**28.7 ± 2.9** ^*^	**35.7 ± 1.4** ^*^	11 ± 0.9	**6.3 ± 1.5** ^*^	**41.3 ± 8.7** ^*^	**3.4 ± 1.3** ^*^	55.1 ± 9.3	**257.9 ± 61.2** ^*^	**0.1 ± 0.04** ^*^	16.8 ± 0.8
0–4 years	5.1 ± 0.5	41.7 ± 4.4	15.1 ± 1.8	80.4 ± 7.3	29.2 ± 3.3	36.2 ± 1.3	11 ± 0.9	6.6 ± 2	42 ± 8.1	3.6 ± 1.1	54.2 ± 8.8	245.1 ± 62.8	0.1 ± 0.04	16.7 ± 0.7
5–14 years	5 ± 0.5	41.6 ± 3.5	15.1 ± 1.3	82.1 ± 5.8	29.8 ± 2.6	36.2 ± 1.3	10.9 ± 0.7	6.8 ± 1.8	42.6 ± 9.1	3.5 ± 1.2	53.7 ± 9.7	240.2 ± 51	0.1 ± 0.03	16.9 ± 0.7
> 15 years	5.1 ± 0.5	41.8 ± 3.7	15.1 ± 1.4	82.3 ± 7.6	29.9 ± 3.3	36.3 ± 1.4	10.9 ± 0.9	7.2 ± 1.8	40.6 ± 9.3	3.4 ± 1.2	55.8 ± 9.8	235.2 ± 53.5	0.01 ± 0.3	16.9 ± 0.8

With the decreasing age of onset smoking in subjects under twenty years old, the RBC count, MCV, MCH, and WBC count increased; also, lymphocytes statistically reduced in this group. However, there was a similar result for monocyte but not significant. The participants with onset age smoking above thirty had a higher level of the PLT count compared with non-smokers and other smoker age groups. Also, the heavy smoker group had higher HGB, HCT, MCH, MCHC, MCV, WBC count, and GR%; also, this group had dramatically lower levels of lymphocytes and monocytes. Increased smoking duration statistically leveled up HCT, HGB, MCV, MCH, MCHC, and WBC count. On the other hand, increased smoking duration decreased values for the lymphocytes and PLT count significantly.

Table 3. Presented the linear regression analysis between five smoking categories and blood parameters. After adjusting for age, gender, physical activity, BMI, CVD history, use of alcohol, HEI, SBP, and DBP, the result illustrated that current smokers had a significantly positive correlation with increased the level of HCT, HGB, MCHC, MCH, MCV, and WBC count. Also, current smokers elevated the level of RDW, PLT count, and GR% but were insignificant. Besides, the relationship between current smokers and RBC count, lymphocyte, and monocyte were negative.

**Table 3:** The multivariable linear regression between smoking categories and blood parameters

Variables		Blood Cell Parameters						
		**RBC count** **β coefficient** **(95% CI)**	**HCT** **β coefficient** **(95% CI)**	**HGB** **β coefficient** **(95% CI)**	**MCV** **β coefficient** **(95% CI)**	**MCH** **β coefficient** **(95% CI)**	**MCHC** **β coefficient** **(95% CI)**	RDW β coefficient (95% CI)
**Smoking** **status**	No	Reference						
	current	-0.02 (-0.059_0.015)	0.**35** **(0.105_0.608)**	**0.30** **(0.207_0.403)**	**1.12** **(0.594_1.648)**	**0.74** **(0.518_0.972)**	**0.40** **(0.296_.514)**	0.01 (-0.059_0.082)
	former	-0.008 (-0.049_0.033)	0.13 (-0.139_0.410)	**0.10** **(0.001_0.216)**	0.35 (-0.223_0.930)	0.235 (-0.012_0.484)	**0.13** **(0.016_0.255)**	-0.028 (-0.105_0.049)
	passive	0.009 (-0.014_0.033)	0.057 (-0.101_.216)	**0.06** **(0.008_.131)**	− 0.056 (-0.388_0.276)	0.07 (-0.064_0.222)	**0.11** **(.049_ 0.187)**	− 0.01 (-0.058_0.031)
**Second hand smoke in current smokers**	No							
	< 2 h	0.004 (-0.020_ 0.028)	0.05 (-0.11_0.220)	0.05 (-0.009_0.119)	0.02 (-0.322_0.378)	0.08 (-0.067_0.234)	**0.083** **(0.009_0.157)**	-0.02 (-0.070_ 0.026)
	2–5 h	0.02 (-0.015_0.072)	0.07 (-.224_.372)	**0.12** **(0.009_0.242)**	-0.35 (-.988_.273)	0.06 (-0.206_0.337)	**0.24** **(0.110_0.375)**	0.02 (-0.057_0.205)
	> 5 h	0.035 (-0.057_0.128)	-0.10 (-0.727_0.527)	0.07 (-0.168_0.320)	-0.76 (-2.09_0.559)	-0.06 (-0.639_0.503)	0.25 (-0.020_0.536)	0.02 (-0.160_0.205)
**Regular age of onset smoke**	No	Reference						
	< 20 years	-0.02 (-0.083_0.026)	**0.57** **(.212_.935)**	**0.38** **(0.246_0.528)**	**1.75** **(0.983_2.535)**	**0.97** **(0.647_1.309)**	**0.40** **(0.249_0.564)**	-0.02 (-0.132_0.074)
	21–29 years	-0.02 (-0.079_0.031)	0.34 (-0.023_0.710)	**0.31** **(0.174_0.460)**	**1.16** **(0.373_ 1.946)**	**0.78** **(0.450_1.121)**	**0.44** **(0.287_0.606)**	0.04 (-0.061_0.148)
	> 30 years	-0.02 (-0.102_0.046)	0.08 (-0.407_ 0.576)	**0.21** **(0.027_0.411)**	0.41 (-0.637_1.473)	**0.49** **(0.048_0.949)**	**0.42** **(0.210_0.638)**	-0.04 (-0.186_0.095)
**Smoking intensity in current smokers**	No	References						
	Light	-0.03 (-0.094_ -0.009)	0.004 (-0.398_0.406)	0.12 (-0.032_0.281)	0.54 (-0.314_1.413)	**0.43** **(0.063_0.799)**	**0.26** **(0.092_ 0.442)**	-0.02 (-0.136_0.094)
	Moderate	-0.009 (-0.085_0.066)	0.272 (-0.230_0.775)	**0.23** **(0.041_0.433)**	0.97 (-0.102_ 2.057)	**0.62** **(0.160_ 1.08)**	**0.31** **(0.099_0.537)**	0.01 (-0.124_ 0.164)
	Heavy	-0.028 (-0.079_0.022)	**0.67** **(0.335_ 1.00)**	**0.49** **(0.359_0.621)**	**1.8** **(1.083_ 2.529)**	**1.117** **(0.809_1.425)**	**0.57** **(0.425_0.718)**	-0.002 (-0.099_0.094)
**Smoking duration**	N0	Reference						
	0–4 years	0.10 (-.025_.235)	0.70 (-0.157_1.56)	**0.45** **(0.119_0.792)**	-0.49 (-2.34_1.35)	0.19 (-0.594_0.984)	**0.41** **(0.379_.788)**	0.06 (-0.183_0.311)
	5–14 years	**-0.09** **(-0.026_-0.070)**	0.03 (-0.56_ 0.632)	0.20 (-0.033_0.434)	**1.47** **(0.189_2.76)**	**0.87** **(0.325_1.423)**	**0.43** **(0.170_.693)**	-0.06 (-0.235_0.109)
	> 15 years	-0.02 (-0.070_0.016)	**0.41** **(0.126_0.699)**	**0.33** **(0.223_0.447)**	**1.35** **(0.735_1.96)**	**0.84** **(0.580_1.104)**	**0.42** **(0.303_0.552)**	-0.0003 (-0.082_0.081)
**Smoking** **status**		**WBC2** **β coefficient** **(95% CI)**	**LYM** **β coefficient** **(95% CI)**	**MONO** **β coefficient** **(95% CI)**	**GR%** **β coefficient** **(95% CI)**	**PLT count** **β coefficient** **(95% CI)**	**PDW** **β coefficient** **(95% CI)**	PCT β coefficient (95% CI)
	No	Reference						
	current	**1.06** **(0.951_1.181)**	-0.31 (-0.970_0.350)	-0.13 (-0.229_-0.040)	0.43 (-0.269_ 1.139)	**1.56** **(-2.841_ 5.965)**	-0.05 (-0.113_0.007)	-0.005 (-0.023_0.012)
	former	0.018 (-.107_.145)	-0.22 (-.943_.502)	-0.006 (-0.109_0.097)	0.20 (-.570_.971)	**-1.406** **(-6.227_3.414)**	-0.01 (-0.083_0.049)	0.023 (0.003_0.043)
	passive	0.01 (-0.057_0.087)	-0.10 (-.521_.312)	**0.03** **(-0.021_0.098)**	0.03 (-.408_.480)	**0.604** **(-2.174_3.383)**	**0.005** **(-0.032_0.044)**	0.001 (-0.010_0.012)
**Second hand smoke in current smokers**	No							
	< 2 h	0.01 (-0.059_0.089)	-0.21 (-0.651_0.229)	0.03 (-0.030_0.096)	0.14 (-0.328_0.610)	-0.94 (-3.942_2.052)	0.003 (-0.036_0.043)	-0.00001 (-0.002_0.002)
	2–5 h	-0.002 (-0.136_.131)	0.09 (-0.698_0.887)	0.04 (-0.066_0.162)	-0.14 (-.991_.698)	4.77 (-0.620_10.167)	0.04 (-0.027_0.116)	0.004 (0.0001_0.008)
	> 5 h	0.16 (-.114_.448)	0.98 (-.679_2.65)	0.05 (-0.185_0.294)	-1.04 (-2.819_ 0.735)	13.3 (1.97_24.669)	-0.109 (-0.261_0.042)	0.01 (0.004_0.022)
**Regular age of onset smoke**	No	Reference						
	< 20	**1.24** **(1.073_ 1.422)**	**-1.00** **(-1.980_-0.034)**	**-0.19** **(-0.330_-0.052)**	**1.19** **(0.161_2.234)**	0.06 (-6.183_ 6.307)	0.001 (-0.003_0.006)	0.01 (-0.073_0.101)
	21–29	**1.078** **(0.901_1.254)**	0.138 (-0.847_1.124)	-0.121 (-09.262_0.019)	-0.01 (-1.066_ 1.034)	2.59 (-3.732_ 8.927)	0.004 (-0.0005_0.009)	-0.089 (-0.178_ -0.0001)
	> 3 − 0	**0.68** **(0.443_0.917)**	0.21 (-1.111_1.535)	-0.096 (-0.285_0.093)	-0.11 (-1.526_ 1.293)	3.03 (-5.45_11.532)	0.003 (-0.002_0.010)	-0.07 (-0.194_0.044)
**Smoking intensity in current smokers**	No	Reference						
	Light	**0.30** **(0.117_0.501)**	0.79 (-0.284_1.879)	-0.02 (-0.183_0.126)	-0.76 (-1.92_0.384)	-7.48 (-14.43_0.540)	-0.004 (-0.009_0.0008)	-0.02 (-.123_0.072)
	Moderate	**1.11** **(0.878_1.35)**	0.79 (-0.56_2.14)	-0.04 (-0.24_0.144)	-0.74 (-2.18_0.69)	7.25 (-1.42_ 15.94)	**0.007** **(0.001_0.014)**	0.03 (-0.089_0.154)
	Heavy	**1.542** **(1.381_1.702)**	**-1.40** **(-2.31_-0.500)**	**-0.25** **(-0.381_-0.122)**	**1.65** **(0.692_2.622)**	**5.72** **(-0.085_11.541)**	**0.006** **(0.001_0.010)**	-0.08 (-0.162_0.0007)
**Smoking duration**	N0	Reference						
	0–4 years	**0.41** **(-0.004_0.826)**	0.68 (-1.63_ 3.01)	0.05 (-0.279_0.384)	-0.74 (-3.21_1.72)	2.03 (-12.8_16.93)	0.002 (-0.008_ 0.013)	-0.16 (-0.37_0.040)
	5–14 years	**0.62** **(.339_.917)**	1.147 (-.467_ 2.761)	0.01 (-.220_.241)	-1.15 (-2.87_.562)	-0.119 (-10.48_10.24)	-0.0005 (-0.008_0.007)	-0.004 (-0.1500_0.141)
	> 15 years	**1.18** **(1.048_1.324)**	-0.59 (-1.36_0.174)	**-0.18** **(-0.291_-0.072)**	0.778 (-0.042_1.59)	1.99 (-2.95_6.93)	0.003 (-0.0001_0.007)	-0.038 (-0.107_0.031)

As shown in Table 3. In secondhand smokers, there was a reverse relationship between exposure time and MCV and MCHC; therefore, the blood levels of these parameters decreased but not statistically. Also, lymphocyte, monocyte, and PCT were raised, and the relation for PCT was significant. Versus, RDW, and MCHC positively related to smoking cigarette exposure and elevated with increasing smoking exposure time. With the decreasing age of onset of smoking, HCT (0.57), HGB (0.38), MCV (1.75), and MCH (0.97) have significantly increased in the under-20s. In contrast, with those under twenty age of onset of smoking, the levels of lymphocyte (-1.0) and monocyte (-0.19) statistically had negative relation. Heavy smoking positively affected RBC parameters (including HCT, HGB, MCV, MCH, and MCHC), WBC indices (WBC count, lymphocyte, monocyte, and GR %), and PLT count and raised these parameters statistically. Increasing cigarette duration use had a significant positive relationship with HCT, MCV, MCH, MCHC, and WBC count. Also, the relation between PLT count (1.99) and GR % (0.77) and duration use time was positive but not significant. However, the association was negative on lymphocytes and monocyte.

**Fig. 1:**
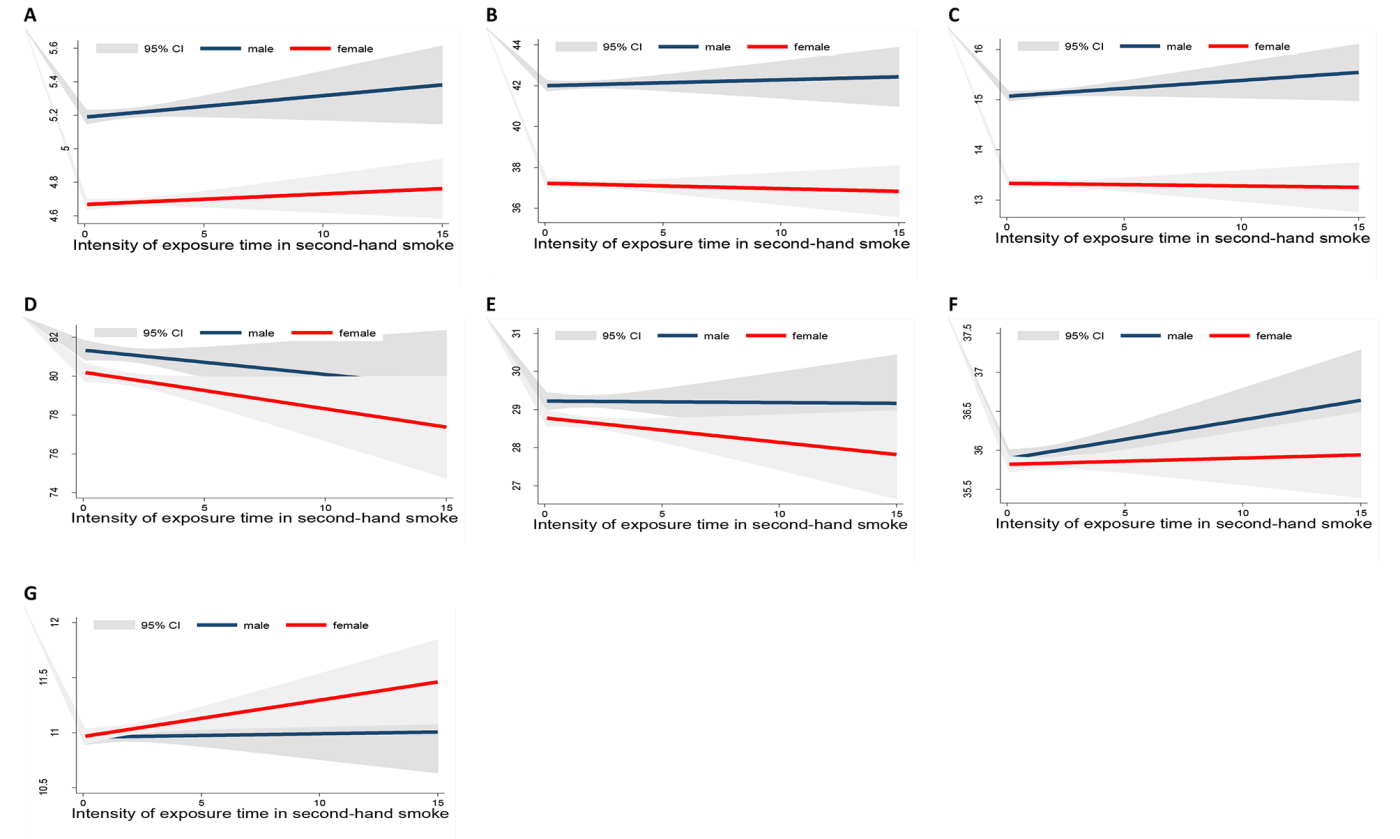
Linear fit plots with confidence intervals of the relationship between the intensity of exposure time in secondhand smoke and RBC indices based on gender

**Fig. 2:**
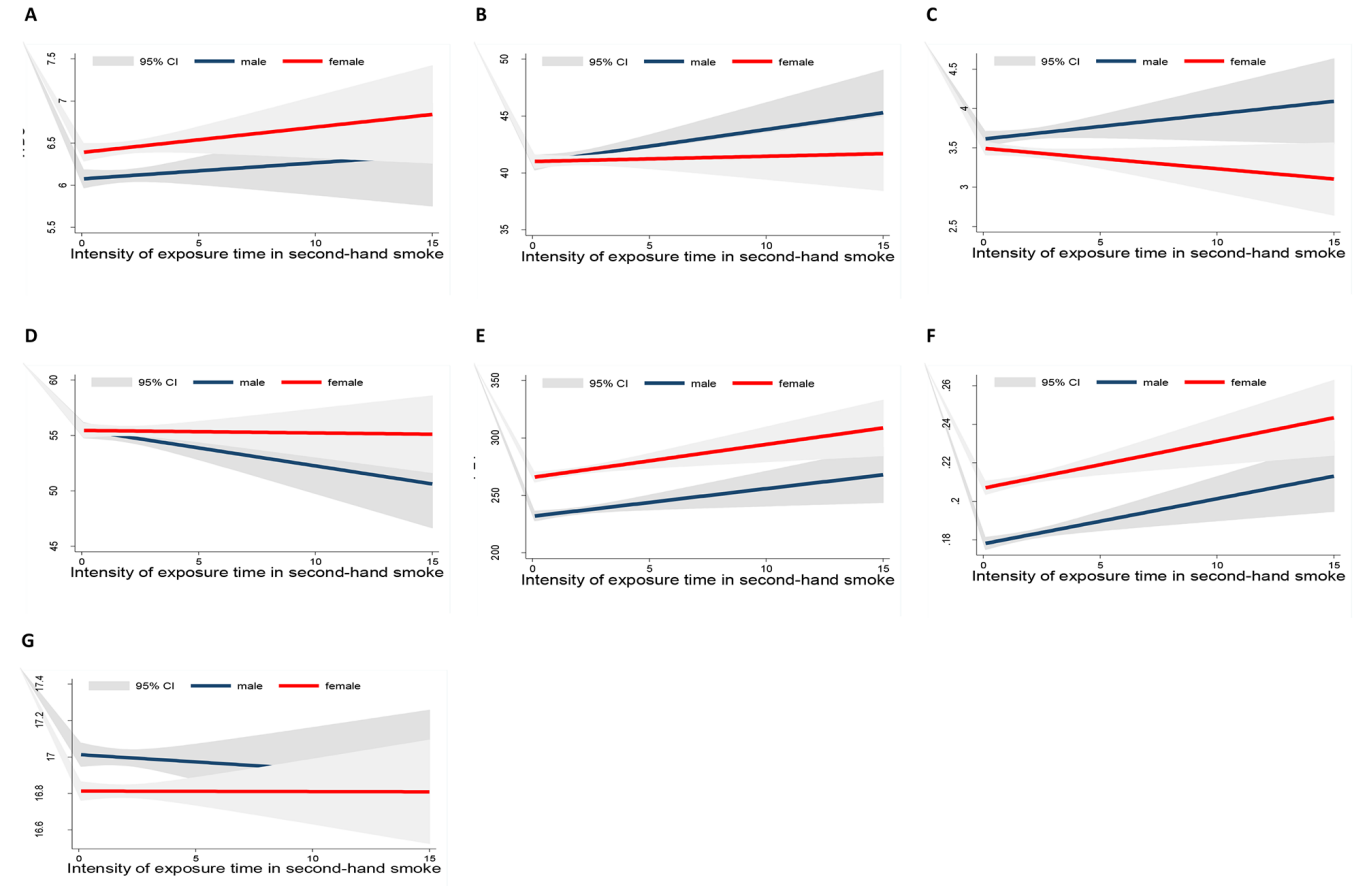
Linear fit plots with confidence intervals of the relationship between the intensity of exposure time in secondhand smoke and the WBC and PLT indices based on gender

In Figs. 1 and 2, we assessed the relationship between the intensity of smoking exposure time based on gender with fit plot linear prediction. The result showed dramatic differences in WBC count, GR%, PLT count, and PDW between male and female populations. In female subjects, the levels of these parameters through the increasing duration exposure time are raised.

## Discussion

The results of the current investigation represent, the prevalence of current, former, and passive smokers was 12.2, 8.9, and 38%, respectively. The highest number of smokers, current (92.1%) and former (83.3%), was in the male population, and the number of females was higher in the passive smoker group (58.1%). The mean BMI was dramatically lower in current smokers than in other groups. Also, the mean BMI of former smokers was near non-smokers. Our study provides similar results consistent with other population-based investigations in the context of associations between smoking and BMI. Current results supported by follow-up studies illustrated that the BMI of former smokers after quitting cigarettes enhanced to the same level as those who had never smoked [17–19]. However, the relation between smoking and BMI is remained unknown [20] but suggested that smoking and nicotine could reduce weight by increasing energy utilization and suppressing appetite [20].

The evidence represented that smokers with higher consumption of alcohol had a poor diet and a lower intake of vitamins such as C and E, beta carotene, and polyunsaturated fat [21]. The current study obtained similar results. Smokers have a higher rate of lower HEI and alcohol consumption.

Regarding blood parameters, current smokers had higher RBC parameters (RBC count, HGB, HCT, MCV, MCH, and MCHC). Also, this pattern repeats with raising the intensity of smoking in current smokers (heavy smokers) and the duration time of using cigarettes (over 15 years). Our results are in line with Pedersen et al. that demonstrated smoking causes increased HCT, HGB, and MCV in smokers compared with non-smokers. However, Aldosari et al. in Saudi Arabia did not find different RBC counts, HGB, and MCV between smokers and non-smokers [6]. Further, in multivariable linear regression analysis in Table 3, after adjusting for age, gender, BMI, physical activity, and HEI, we found that current and heavy smokers and smoking cigarettes over 15 years’ groups had a maximum positive significant correlation with HCT, HGB, MCV, MCH, and MCHC. Tobacco generates more than five thousand chemical compounds, including gases and particulates like nicotine, nitrosamine, formaldehyde, and carbon monoxide, and hydrogen cyanide [3]. In alveolar capillaries, carbon monoxide(CO) with a binding ability of 200–250 times greater than oxygen can bind firmly to the HGB, forming carboxyhemoglobin and leading to hypoxia in tissues subsequently leading to high values of HGB and RBC count in smokers. We demonstrated that individuals with heavy smoking and using smoked over 15 years groups had statistically higher HGB and HCT. In other words, increased hemoglobin levels by creating polycythemia and decreasing blood speed could elevate the risk of intravascular clotting, coronary vascular resistance, and incidence of thrombosis [22, 23]. Furthermore, smoking cigarettes establishes a condition of combined polycythemia and chronic hypoxia, which causes RBC production to ascend due to an increased carboxyhemoglobin and consequently leads to plasma volume reduction [22]. In our study, heavy and using more than 15 years smokers significantly had higher SBP which might occur due to raising RBC and HTC levels that puts extra strain on the vascular system and increases the risk of cardiovascular disease [24]. Also, in categories of age-onset under 20 and smokers over 15 years, current, and heavy smokers, the levels of MCV raised dramatically compared to other groups. Moreover, we found a negative relationship between smokers category groups and RBC count. Change in MCV is probably derived from the effects of CO hypoxia on the rise in RBC count. Since the demand for raising RBC cannot be met because of the cyanide-induced diversion of vitamin B12, consequently leads to a change in RBC morphology [25]. In the current study, smokers age-onset under 20 and smokers over 15 years, current and heavy smokers had a significantly higher WBC count, GR%, and lower lymphocyte count. This reduction in smokers over 15 years and heavy smokers was significant. Although in the study of Pedersen and SHIPA et al., along with an increase in WBC count, the lymphocyte count was also higher in smokers than non-smokers [10, 22]. Several mechanisms can explain the rise in WBC count in smokers.1. The effect of nicotine on the adrenal medulla leads to releasing catecholamines, which might raise the WBC count by changing the leukocyte distribution from the marginal pool into the circulating pool [26]. 2. Free radicals produced by cigarettes are consistently correlated with different inflammatory indices like WBC parameters, and acute-phase proteins such as C-reactive protein. Also, bronchial tract inflammation in smokers may increase leukocytes [26, 27]. The impact of smoking on different types of leukocytes is remained controversial [28]. Although the current investigation showed a lower level of lymphocyte count in smokers, a considerable increment in the rate of granulocytes (GR %) could indicate an increase in other types of white blood cells such as neutrophils, basophils, and eosinophils, as Pedersen et al. illustrated that smoking has a positive effect on the neutrophil count [10].

In context with PLT, we found the mean level of PLT count in passive smokers was higher statistically than in other groups. Moreover, with increasing smoking exposure time in secondhand smokers, the level of PLT count and PCT dramatically raised. In the multivariable regression model, current smokers had the most positive relationship with PLT count. Also, passive smokers had a significant positive relation with PLT count, but the relationship was negative in former smokers. Exposure to cigarettes also positively affected PLT and PCT but was not significant.

Another noteworthy point was that smoke intensity rather than smoking duration time had more impact on PLT count. So, in heavy smokers, 5.72 fold-up the PLT count, but smoking over 15 years just 1.99 fold-up increased the level up PLT count. Passive smoking is a potential risk factor for coronary artery disease. The involved mechanisms for passive smokers are the same as active smokers. Nitric oxide is decreased by free radicals derived from oxygen, which is produced by cigarette components found in the tar and gas phases. Consequently, vasodilation of vessels decreases, and the risk of incorporation of PLT and leukocytes into the vascular septum and the formation of vascular plaques increases [5]. The metal ingredients of cigarettes such as Cadmium [29, 30] can increase the protein oxidation of cells that contribute to sediment in the aortic wall and disposes of smokers and passive smokers to MI and hypertension [30]. In line with the explanations, and according to our studies, the prevalence incidence of CVD was higher in passive smokers. Although the mean SBP was more elevated in former smokers, the high prevalence rate of HTN belongs to the inactive smoker group. Also, assessment participants by gender, with increasing exposure time to cigarette smoke, the level of WBC count, GR%, PLT count, and RDW were increased in females dramatically. Women have a more robust inflammatory system than men. Therefore, women are more susceptible to chronic inflammatory and immune diseases such as cystic fibrosis, asthma, and lupus. Studies have shown that women had very high C-reactive protein and neutrophil levels and erythrocyte sedimentation rate in infectious diseases [5, 31]. However, we did not assess white blood cells separately in the present study.

Nevertheless, an increase in GR% and a decrease in lymphocyte and monocyte levels could indicate an increase in neutrophils in the female population. The role of inflammation in heart disease may also indicate that females exposed to secondhand smoke have a higher chance of developing CVD among people exposed to secondhand smoke.

The current study has some strengths and limitations that must be considered. To the lead of our information, the present investigation is the first study performed on a large-scale population of Iranians that analyzes the relationship between blood parameters and different smoking cigarettes. In this study, we tried to categorize and examine various groups of smokers to get a better view of smoking in the Iranian population. For this reason, the results of the study can be a basis for future studies in Iran and the countries of the Middle East region. Likewise, our findings could be beneficial information for health policymakers to design and execute effective preventative programs against tobacco use to protect the Iranian population. Regardless, there are limitations to the present study. We tried to exclude all participants with underlying diseases and receive drugs that could affect the investigation results. Still, there are medications used by the participants whose effects we have not investigated and controlled. Besides, opium consumption was obtained by the individual’s self-report. So, we could not collect acceptable data for analysis. Also, we could not find similar studies in this field on the Iranian population to better compare the obtained results. In addition, follow-up of individuals is necessary to achieve more complete results and a better understanding of the effect of smoking on the hematopoiesis end product.

## Conclusion

In the present study, the association between smoking in different categories and blood parameters was evaluated. The results showed that smoking positively correlated with RBC indices (RBC count, HCT, HGB, MCV, MCH, and MCHC), WBC count, GR%, and PLT count. The levels of these parameters were considerably higher in smokers compared with non-smokers; on the other hand, lymphocytes and monocytes were negatively related to smoking. However, the mean of these factors was more elevated in active smokers than in other groups. Also, passive smokers had a higher PLT count and PCT levels, and the prevalence of CVD and HTN was more elevated in this group.

Additionally, we found that the WBC count, GR%, PLT count, PCT, and RDW had dramatically increased through an increased smoke time exposure in female passive smokers which might indicate females are more vulnerable than men to cigarette smoke. Since there was a positive correlation between cigarette smoke and PLT indices in the passive smoker, we suggest that the screening test for coronary artery disease must be considered in this population along with current smokers and whereas most secondhand smoke population usually belongs to females, for more clarity on the results of secondhand smoke, more investigation on a males population recommend.

## Data Availability

The data utilized are available in the current examination based on reasonable request from the corresponding author.
